# An intervention to provide nutritional care for people living with dementia at home receiving home care (TOMATO): study protocol for a single-arm feasibility study

**DOI:** 10.1186/s40814-025-01722-5

**Published:** 2025-11-21

**Authors:** Gladys Yinusa, Claire Surr, Sarah Thomas, Lee-Ann Fenge, Daniel Howdon, John Major, Michelle Heward, Gordon Taylor, Helen Knight, Jane Townson, Jane Murphy

**Affiliations:** 1 Ageing and Dementia Research Centre, Faculty of Health and Social Sciences, Bournemouth University, Bournemouth, UK; 2 Centre For Dementia Research, Leeds Beckett University, Leeds, UK; 3 Clinical Research Unit, Faculty of Health and Social Sciences, Bournemouth University, Bournemouth, UK; 4 Centre for Seldom Heard Voices, Faculty of Health and Social Sciences, Bournemouth University, Bournemouth, UK; 5 Academic Unit of Health Economics, University of Leeds, Leeds, UK; 6 Research Design Service for the Southwest, University of Exeter, Exeter, UK; 7 Leeds Community Healthcare NHS Trust, Leeds, UK; 8 Homecare Association, London, UK; 9 NIHR Applied Research Collaboration Wessex, Southampton, UK

**Keywords:** Dementia, Alzheimer’s disease, Malnutrition, Undernutrition, Nutritional Care, Eating and Drinking, Home Care, Feasibility study, Intervention, Ageing

## Abstract

**Background:**

In the UK, over 980,000 people are living with dementia, and two-thirds of them live in their own homes. Up to 60% of this population is estimated to be at risk of or already experiencing malnutrition, with 45% facing significant weight loss. As dementia progresses, ensuring that people eat and drink well becomes challenging. Many families affected by dementia access home care services, with home care professionals playing a vital role in supporting and enhancing overall quality of life. Training in identifying nutritional problems and supporting family carers to prevent malnutrition is an identified research need; however, research on the contribution of home care professionals in this area is limited. This study aims to assess the feasibility and acceptability of a nutritional intervention for people living with dementia receiving home care from the perspectives of people with dementia, family carers (dyads), and home care professionals (including home care managers).

**Method:**

This is a mixed-method single-arm feasibility study of a nutrition intervention with embedded process evaluation. Thirty-two participants living with dementia and their carers (dyads) will be recruited from home care organisations providing services for older adults across the South, Midlands, and North of England. The intervention comprises a nutritional awareness training session for home care workers, combined with educational resources for home care professionals, family carers, and friends. It is based on a model of person-centred nutritional care and will be delivered by trained home care professionals in the homes of participating dyads over 4 months. Outcome measures will be collected at baseline and at 4 months.

Analyses will be descriptive and centred on the feasibility and acceptability of the interventions and study procedures. Key feasibility outcomes will include the rate of participant recruitment and dropout, and the percentage of home care staff who adhere to the intervention schedule (setting at least four actions in response to using the resources). Quantitative data analysis will primarily involve descriptive statistics. Acceptability of the intervention will be determined through in-depth semi-structured qualitative interviews conducted with a subsample of participants dyads and home care professionals. An embedded process evaluation will assess intervention implementation, capturing barriers and facilitators through participant interviews.

**Discussion:**

Findings from this study will help inform the development and implementation of a future RCT, should this nutrition intervention be feasible.

**Trial registration:**

NCT05866094.

**Supplementary Information:**

The online version contains supplementary material available at 10.1186/s40814-025-01722-5.

## Background

Over 980,000 people in the UK live with dementia, and this number is predicted to rise to over 2 million by 2050 [1], imposing an annual cost of £26 billion on the UK economy [2]. Poor nutrition significantly contributes to ill-health and stress for people living with dementia and their carers [3]. The management of eating and drinking difficulties has been identified as a research priority [4]. Two-thirds of people with dementia live in their own homes [5], with estimates indicating between 14% [6] and 60% [7, 8] are with or at risk of malnutrition and 20–45% experiencing significant weight loss [3].

While health care professionals like district nurses, practice nurses, and general practitioners (GPs) offer treatment and care to people with dementia in the community, nutritional care may not be prioritised until the point of deterioration, despite the National Institute for Health and Clinical Excellence (NICE) guidance and quality standards [9, 10]. Health care professionals face various barriers to providing nutritional care, including brief and infrequent contacts often related to other conditions (e.g. diabetes clinic, blood tests), organisational culture, lack of fit-for-purpose screening tools, limited knowledge and skills, and poor communication between services [11]. As a result, health care professionals may not be best positioned to identify and manage nutrition-related problems, particularly at an early stage. The responsibility for day-to-day nutritional management predominantly falls on family carers, who often lack information and resources, thereby increasing the risk of nutritional decline for the person living with dementia [12].

There is a need for interventions to improve nutritional care for people with dementia living at home. Many families affected by dementia rely on home/domiciliary care services, with over 400,000 accessing these services across the UK [13]. Most home care, including local authority-funded services, is provided by the independent sector. Home care professionals typically see care recipients more frequently and for longer than health care professionals, and their role may involve supporting eating, drinking, and meal preparation. They are potentially well-placed to identify weight loss, eating and drinking difficulties, reduced nutritional intake, the risk of malnutrition, and to support family carers in delivering nutrition-based interventions. Studies have highlighted the need for home care professionals to receive training and support in identifying nutritional problems and supporting family carers around managing eating, drinking, and preventing malnutrition [14, 15]. Appropriate nutrition and hydration care is a component of the core principles and standards for supporting people living with dementia in health and adult social care [16]. However, research on the contribution that home care professionals might make in this area is limited.

Targeting interventions at home care professionals and family-carer dyads, with input from appropriate health care professionals, may provide a proactive and wide-reaching approach to nutritional assessment and management. Malnutrition and unintentional weight loss in people living with dementia, resulting from reduced appetite and difficulties with eating and swallowing [17, 18], significantly contributes to physical and cognitive decline, reduced quality of life, increased health care utilisation [18, 19], and substantial Health and Social Care expenditure (approximately £23.5 billion annually) [20]. Early identification of nutrition-related health risks and intervening via referral to nutrition support and advice from health care professionals can prevent deterioration and potentially reduce associated health care service costs for treating malnutrition. Early intervention may also alleviate the stress and burden that eating and drinking difficulties impose on family carers, potentially reducing or delaying admission to residential care [21]. Developing an intervention for home care professionals and family carers, aimed at supporting people with dementia living at home, is a crucial step towards improved care and outcomes. This initiative aligns directly with NHS priorities and the NHS Long Term Plan [22] to develop new and effective integrated pathways for better care across health and social care systems.

The Health and Social Care Act 2008 (Regulated Activities) Regulations 2014 (Regulation 14), enforced by the Care Quality Commission (CQC) [23], mandates that provider care organisations assess individuals’ nutritional needs and provide appropriate nutrition and hydration to meet those needs. This regulation forms part of the overarching requirement for providers to deliver safe, effective, and compassionate care. Despite this standard, there remains a need to enhance the delivery of nutritional care. A scoping review (35 studies) on managing the nutritional care of people with dementia at home [24] revealed nutrition-related difficulties leading to inadequate or unbalanced dietary intake and increased malnutrition risks at all stages of the disease. While some beneficial outcomes for nutritional status were observed in intervention groups, the authors identified a need for further research to develop and test acceptable and effective individualised nutritional interventions. In the home care context, a qualitative study [15] exploring the experiences of both health care professionals and home care professionals highlighted the challenges of providing effective care and support, emphasising limited time when supporting people living with dementia and poor knowledge of appropriate food and drink choices. However, the study identified that collaboration between home care professionals and family carers could improve care outcomes. Two European randomised controlled trials (RCTs) [25, 26] involving a nurse- or multi-disciplinary team delivered nutrition intervention for older people at home reported positive effects on quality of life and delayed nutritional decline. In line with recommendations from our research in the care home context [27], the scoping review [24] similarly advocated a whole-systems approach to delivering nutritional care for people living with dementia receiving home care.

This protocol reports the current study (TOMATO: nuTritiOn and deMentia AT hOme), designed with the primary aim to assess the feasibility and acceptability of a nutritional intervention adapted for people living with dementia receiving home care. The study will explore the perspectives of people with dementia, their family carers, and home care professionals (including home care managers).

## Nutritional intervention adaptation

This section briefly outlines the adaptation of the nutritional intervention. The intervention was adapted from our evidence-based care home resource [27, 28] and included dementia training for care staff, based on good practice and as identified in previous research [29], which addressed similar learning, development, and language needs of the workforce.

We utilised the ‘ADAPT guidance’ for adapting interventions to new contexts to tailor the intervention to the home care setting [30]. Insights gathered from qualitative interviews (conducted individually and in pairs) with people living with dementia, their family carers, and homecare professionals informed the refinement of the intervention. This provided a framework based on the model of person-centred nutritional care underpinned by six core factors, and the key principles are summarised in the logic model (see Additional file 2).

Following the Behaviour Change Wheel (BCW) systematically [31], we iteratively conducted the COM-B (Capability, Opportunity and Motivation Behavior) mapping for the nutrition intervention. In line with the recommendations for using the BCW in practice [32, 33], the Acceptability, Practicability, Effectiveness/cost-effectiveness, Affordability, Safety/side-effects, Equity (APEASE) criteria were applied during initial judgments to decide which intervention functions, policy categories and behaviour change techniques (BCTs) were most appropriate.

The mapping led to the identification of five of the six COM-B constructs (Psychological Capability, Physical capability, Social Opportunity, Physical Opportunity and Reflective Motivation), five components of the intervention functions (Education, Training, Enablement, Environmental Restructuring, Modelling), three policy categories (Communication/Marketing, Guidelines, and Environmental/Social Planning) and 12 associated Behaviour Change Techniques (BCTs) based on BCT Taxonomy version 1 [34] [Problem solving, Goal setting (outcome), Action planning, Instruction on how to perform the behaviour, Information about health consequences, Information about social and environmental consequences, Demonstration of the behavior, Prompts and cues, Credible source, Restructuring the physical environment, Restructuring the social environment and Adding objects to the environment]. Comprehensive details about the intervention adaptation process (Phase 1) will be published separately.

Additionally, the intervention adaptation was informed by input from the TOMATO Patient and Public Involvement (PPI) group, with the Project Steering Group (PSG) providing expert and lived experience insight throughout the process. The tailored intervention components consist of home care professionals’ nutrition awareness training and adapted educational resources (detailed and handy guides for home care professionals, family carers and friends) with validated tools (e.g. the Patients Association Nutrition Checklist [35] and simple practical approaches to help improve and support eating and drinking well for people living with dementia receiving home care.

## Objectives

### Primary objectives

1. Assess the acceptability and feasibility of the nutritional intervention including fidelity and adoption and understand the barriers and facilitators to using the intervention.

### Secondary objectives

1. Collect data on the cost and resource use of health and social care services during the delivery of the intervention
2. Evaluate the feasibility and acceptability aspects of the research design and procedures (e.g. screening, consent processes, recruitment and attrition rates and potential outcome measures) to help inform the planning and design of a future trial.

## Methods

### Study setting and participants

Thirty-two participants with dementia and their carers will be recruited via four to eight home care organisations that provide services for older adults (with an average of 8 dyads per home care provider), across the South, Midlands, and North of England.

We will advertise the opportunity to participate in this study by speaking at forums attended by home care managers and through direct contact with national home care organisations (with franchises) and smaller independent providers, drawing on the network with which the project team already has an established relationship.

We will work together with the participating home care providers (funded privately or by the local authority) across these regions to ensure variability in demographics, diversity, and local services. The nutrition intervention will take place in participants’ own homes.

### Study design

This is a single-arm, non-randomised feasibility study in which participants living with dementia who use home care services will receive the intervention. A mixed-methods approach will be used to assess the feasibility and acceptability of a nutritional intervention for people with dementia living at home, with an embedded process evaluation. This evaluation will collect data on intervention fidelity and implementation, including barriers and facilitators, to contextualise the findings, and inform a future trial.

The design of this protocol follows the Standard Protocol Items Recommendations for Interventional Trials (SPIRIT) 2013 guidelines (see Additional file 1) [36]. Results will be reported in line with the CONsolidated Standards of Reporting Trials (CONSORT) extension for pilot and feasibility studies [37] and the Template for Intervention Description and Replication (TIDieR) checklist (see Additional file 2) [38]. Implementation and evaluation will be guided by Normalisation Process Theory (NPT) [39], which is underpinned by four key constructs: coherence or sense-making, cognitive participation, collective action, and reflexive monitoring. The NPT focuses on the activities that individuals and organisations need to undertake to integrate and normalise new, complex practices into their daily routines. Additionally, the RE-AIM framework for process evaluations and implementation [40] will be used to report outcomes that will guide future trial planning.

This study is registered with the ClinicalTrials.gov database, trial number NCT05866094.

### Patient and public involvement

The research team established a PPI group of eight members, including people living with dementia, family carers, and home care professionals, with a designated lay PPI lead. Together, they contribute valuable insights informed by their lived experience. They will continue to meet online at key stages throughout the study.

Their valuable input will be represented in the core TOMATO research team meetings, informing the project design, ethics documentation and study delivery. The group will also continue to discuss and review the study's progress, research findings and dissemination activities. Additionally, they will be actively involved in the preparation of public-facing dissemination materials.

### Eligibility criteria

The study eligibility criteria are presented in Table 1. The study participants include (1) people living with dementia who are receiving home care; (2) their family/carers, and (3) home care professionals and managers from the participating home care provider organisations.

**Table 1:** Eligibility criteria for people living with dementia, carers and home care professionals

	Inclusion criteria	Exclusion
People living with dementia	• formal diagnosis or functional symptoms associated with probable dementia (assessed by a senior home care staff using the FAST© [41]). • In receipt of home care services • currently at risk of, or experiencing malnutrition (assessed by a senior home care staff using the ‘Patients Association Nutrition Checklist’) [35]. • have family member/friend providing care/support resident in the same house as the person with dementia or living close by. *For interviews, people living with dementia will* • Currently be or have recently been (in last 6 months) at risk of/experienced malnutrition. • Be able to communicate fluently enough in English or provide informed consent with the assistance of a translator or family member to participate. • Be able to recall their experiences of malnutrition.	• At end of life or on an end-of-life care pathway. • Permanently cared for in bed. • Do not have an informal caregiver. • Receiving specialist nutritional support, e.g. feeding via tube.
Carers and home care professionals	• Currently or have recently (in last 6 months) provided care for someone living with dementia at risk of or experiencing malnutrition. • Able to communicate fluently enough in English or provide informed consent to participate with the assistance of a translator or family member.	• Have not recently (in last 6 months) provided care for someone with dementia at risk of/experiencing malnutrition. • Not able to communicate fluently enough in English or provide informed consent.

Functional Assessment Staging Test (FAST) [41] describes seven stages in the progression of Alzheimer’s disease, with good validity and reliability FAST focuses on the ability to perform activities of daily living, delineating the progressive loss of functioning through seven stages (from 1 = normal adult to 7 = severe Alzheimer’s)

### Participant recruitment and timeline

**Fig. 1:**
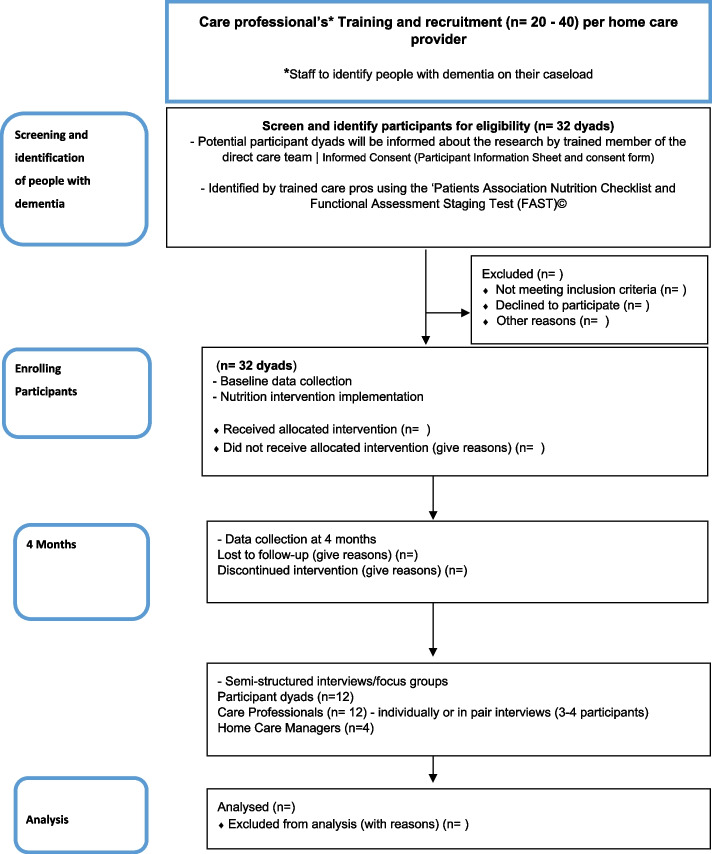
TOMATO study flow diagram

Participants will be recruited via the home care provider organisations as depicted in the study flow diagram (see Fig. 1).

The research team will provide training to the senior home care professionals on how to apply the eligibility criteria using the ‘Patients Association Nutrition Checklist’ [35] to identify potential participants at risk of malnutrition who will then be introduced to the researcher with their permission. Identified dyads will be informed about the research and asked by a senior home care professional if they would be happy to speak to a representative from the research team about taking part.

Identification and initial screening of potential participants living with dementia will be undertaken by a trained senior member of the direct care team from each participating home care provider. The person conducting the initial assessment will complete the screening form in line with the eligibility criteria. The researcher will also complete the form upon confirming eligibility and obtaining informed consent.

Home care professionals fulfilling the eligibility criteria will be identified and approached via a manager or senior member of staff from each participating home care provider. We will recruit additional home care professionals during the intervention to accommodate any loss to follow-up.

### Informed consent

All potential participants—people living with dementia and their family carer dyad, and home care professionals (triad)—will receive Participant Information Sheets (PIS), either directly from the researcher or through the home care provider. These will include a short one-page information leaflet with suitable visuals explaining the project and a consent form. The researcher will only contact eligible participants who have expressed interest and are willing to speak to them about taking part in the study. Trained researchers will explain the study to participants (using the PIS and consent form) and obtain written informed consent from those who volunteer and meet the eligibility criteria (see Table 1).

The researcher will make any formal decisions regarding capacity to consent to take part in the study. Formal assessment of capacity will be conducted by the researcher in accordance with the Mental Capacity Act (2005). If a person living with dementia lacks the capacity to consent, a personal consultee will be approached. A suitable personal consultee will be determined in consultation with the person living with dementia and the family member. If the personal consultee is willing, they will be asked to advise on the person's wishes regarding participation in the study. They will be provided with the consultee information sheet and consultee declaration form. Written informed consent will be obtained using the consultee declaration form.

Participants will have the option to complete the consent form either face-to-face (using the paper consent form) or electronically (eConsent). All participants will be informed that they are free to withdraw from the study at any stage without penalty.

### Sample size

As this is a feasibility study, a formal sample size calculation is not required. We will aim to recruit a purposive sample across a diverse range of participants including socio-demographic, carer relationship and living arrangements, dementia severity and length of dementia caring/working experience. The target sample size is 32 participant dyads (person with dementia and carer) from across the participating home care providers, with the aim of achieving 25 dyads at the 4-month follow-up. This accounts for approximately 20% dropout and provides a 95% exact binomial confidence interval for retention, assuming 32 dyads are enrolled and 80% retained, from 64% to 93%. These align with sample size recommendations and is consistent with those typically indicated for feasibility studies [42].

We will train enough staff to deliver the intervention to ensure that recruited dyads will have at least 75% of their home care visits conducted by trained home care professionals. The number of trained staff will be based on usual working patterns, such as shift patterns, consistency of allocation to people receiving support, agency size, and working logistics, with an anticipated range of 20 to 40 staff per organisation. Table 2 outlines the schedule of enrolment, intervention and assessment.

**Table 2:**
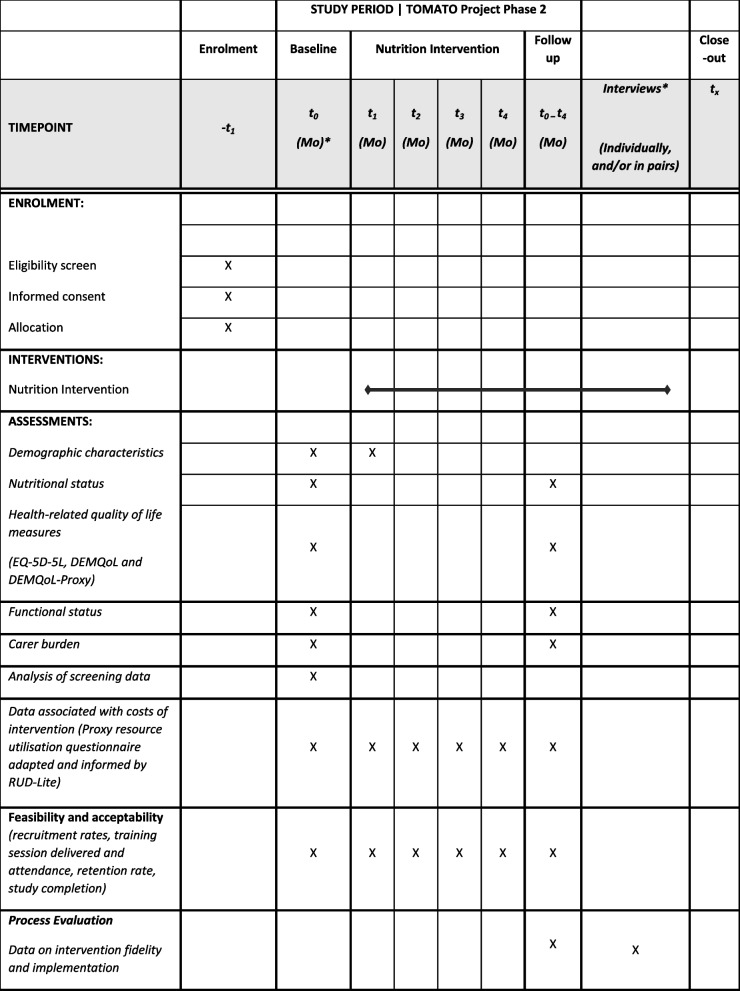
TOMATO project schedule of enrolment, intervention and assessment

### About the adapted nutritional intervention

The tailored nutrition intervention is based on the key principles of the person-centred nutritional care model (see Additional file 3: TOMATO logic model). The nutrition intervention targets home care professionals by delivering nutrition awareness training and providing them with adapted resources. These resources demonstrate various practical ways, including behavioural and environmental aspects, to take action and support people living with dementia at home with eating and drinking.

The intervention will be delivered by trained home care professionals in the homes of the participating dyads over a 4-month period.

#### Home care professional’s training session (face to face and online)

The nutrition awareness training session for home care professionals will be delivered face-to-face or online. Online sessions will be delivered either on individuals’ own devices or via the participating home care organisation’s online platform, depending on preference. The training incorporates presentations using PowerPoint slides and group discussions.

The training covers awareness raising about person-centred nutritional care, how to spot nutritional decline, availability of food and drinks, monitoring and care planning, working together with family carers and friends, enhancing the mealtime experience at home and engaging in activities, ensuring consistency of care and sourcing evidence-based information. This will ensure all staff possess the required knowledge to identify people at nutritional risk and to implement nutritional care plans. The training is expected to last approximately 2 h and will feature case scenarios and group discussions.

Upon completion of the nutritional care awareness level training, all staff will receive a certificate of completion as part of their continuing professional development (CPD). The training session will be conducted by an expert nutritionist-dietitian and trained research team facilitators. Home care professionals who cannot attend the training on the day will have the opportunity to attend a separate session. Table 3 shows details of the intervention, the COM-B components and highlights the training session’s main content and delivery mode.

**Table 3:** Component of the nutrition intervention

Component of the nutrition intervention					
Intervention component and delivery mode	Objectives	Training session *content*		Behaviour Change Wheel (BCW) Intervention Functions	Associated Behaviour Change Techniques (BCTs)
		Topics covered	Key principles		
1.Training session (online and face to face) 2. Guidebook on eating and drinking well at home with dementia, provided in a comprehensive and handy guide format for easy access and reference	1. To raise awareness about nutrition (eating and drinking) for people living with dementia at home 2. To ensure home care professionals have the required knowledge about spotting people at risk of undernutrition and actions to take in response 3. To ensure home care professionals have access to evidence-based information booklet (detailed and handy guide) on eating and drinking well with dementia at home	Availability of food and drinks	How to accommodate for changes in appetite, food preferences and ability with practical suggestions for modifying food, planning menus and improving the presentation of food	Education	5.1. Information about health consequences 5.3 Information about social and environmental consequences 7.1 Prompts/cues 9.1. Credible source
		Monitoring and care planning	Spotting the signs of poor nutrition and using validated tools to identify people likely to be at risk of undernutrition	Training	4.1. Instruction on how to perform the behaviour 6.1 Demonstration of the behaviour
		Relationship: Working together	Practical ways of working together with family members and close friends to improve food and drink intake	Enablement	1.2 Problem solving 1.3. Goal setting (outcome) 1.4 Action planning 7.1 Prompts and cues 12.5 Adding objects to the environment
		The mealtime experience at home and activities	Improving the dining environment and mealtime experience practical activities to stimulate appetite, evoke memories and create a sense of purpose	Environmental restructuring	12.1. Restructuring the physical environment 12.2. Restructuring the social environment 12.5. Adding objects to the environment
		Consistency of care	Improved communication between all those involved with care and the provision and prioritisation of nutrition and hydration	Modelling	6.1 Demonstration of the behaviour
		Provision of trusted information	Access to trusted information, training, resources and current guidelines		

#### Resources: eating and drinking well with dementia at home guide

**Fig. 2:**
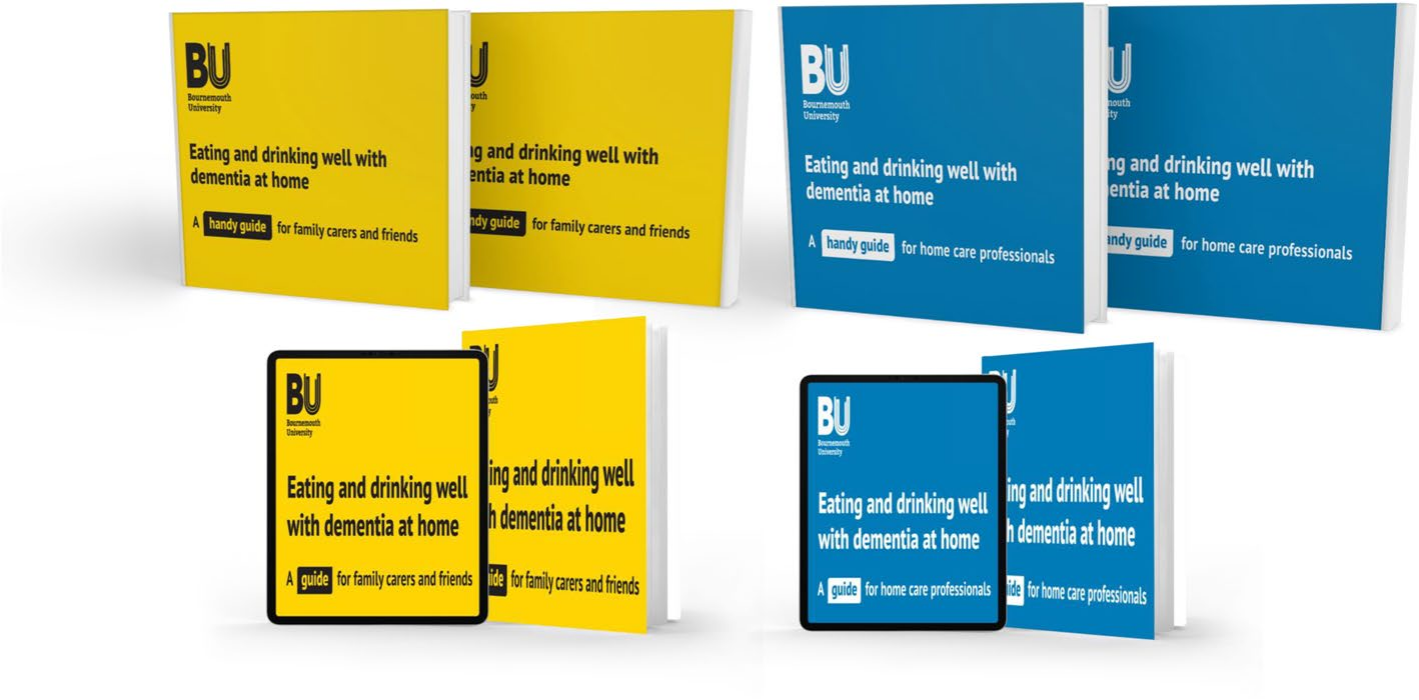
Eating and drinking well with dementia at home resources to be used during the intervention testing

The adapted guides contain detailed information on person-centred nutritional care domains, consistent with the content of the nutrition awareness training. All participating family carers, friends, and home care professionals will receive both the detailed guidebook and a ‘handy’ guide titled Eating and Drinking Well at Home with Dementia, which offers simple approaches and tools to support the nutritional care of people living with dementia. The handy guide, a small-sized booklet, is designed for easy access and reference (see Fig. 2).

#### Additional support

A trained member of the research team and facilitator will contact participating home care professionals via telephone or email to provide additional support and encourage monthly reflection, including the documentation of an action log on the intervention throughout the 4-month intervention period. Peer support will be promoted among trained participating home care professionals, with peer discussions focusing on addressing any challenges faced, as well as on person-centred nutrition care topics covered during the nutrition awareness training and in the resources.

### Outcome measures and data collection

The primary outcome will be an assessment of study feasibility, including acceptability, recruitment and retention, protocol adherence and adaptations, compliance with study procedures including completion of questionnaires, percentage withdrawal, completion of outcomes and assessments, barriers and enablers to intervention delivery. We will relate the evaluation to focused components of feasibility studies [43]. This includes the assessment of acceptability, demand, implementation, practicality, adaptation, integration, and expansion. Secondary outcomes will be collected at baseline and 4-month after the first visit by the home care professional, unless recorded as routine data, at other time intervals. In such cases, all data recorded over the intervention period will be gathered. They will include nutritional status of the person with dementia (body weight, height to calculate Body Mass Index, BMI of < 20 kg/m^2^ as an indicator of undernutrition), quality of life using EQ-5D-5L/EQ-5D-5L-Proxy and DEMQoL/DEMQoL-Proxy [44–46] (as a robustness check and interview administered) and functional status (Deterioration in Daily Living Activities in Dementia) [47] and carer burden using Zarit Burden Interview [48]. Outcome measures were selected based on our logic model and informed by consultation with the PPI and Project Management Group. Table 4 outlines these assessments and strategies for measuring the outcomes of the TOMATO study.

**Table 4:** TOMATO outcome measures

Feasibility parameter	How they will be assessed?		When the measures will be collected? [pre- only, post- only, pre and post intervention]
Recruitment rate	Percentage of participants recruited from participating home care provider		Pre and post
Attrition rate	Percentage of participants who drop out of the study before completion		Pre and post
Intervention Adherence	Percentage of staff who adhere to the intervention schedule (including the completion of the monthly action logs for 4 months)		Post-only
Components of focus			
Acceptability	Semi-structured interviews/focus group with participants		Post-only
Demand	Analysis of screening data Semi-structured interviews/focus group with participants		Post-only
Implementation	Documentary analysis Semi-structured interviews/focus group with participants		Post-only
Practicality	Semi-structured interviews/focus group with participants		Post-only
Adaption	Documentary analysis Semi-structured interviews/focus group with participants		Post-only
Integration	Documentary analysis Semi-structured interviews/focus group with participants		Post-only
Expansion	Documentary analysis Semi-structured interviews/focus group with participants		Post-only
Assessment	Outcome measures	Description	
Participants living with dementia			
Nutritional status Weight-related health risks	Body Mass Index (BMI)	Body weight (kg) and height (m^2^) will be combined to determine BMI (weight (kg)/height (m^2^)). BMI of < 20 kg/m^2^ will be an indicator of undernutrition	Pre and post
Health-related quality of life (HRQoL)	Dementia Quality of Life questionnaire (DEMQoL) and DEMQoL-Proxy	4 level scale across 28 items (DEMQoL) or 31 items (DEMQoL-Proxy) with higher scores indicating better quality of life	Pre and post
	EuroQol 5-Dimension 5-Level (EQ-5D-5L) and EQ-5D-5L-Proxy	The descriptive system assesses health-related quality of life across five domains using a 1–5 level scale, where levels range from 1 (indicating no problems) to 5 (indicating extreme problems). The visual analog scale (VAS) measures quality of life on a scale of 0 to 100, with higher scores indicating better quality of life	Pre and post
Functional status	Deterioration in Daily Living Activities in Dementia) using Modified Barthel Index measures	2-point scale rating with 0 to 20 points. Higher score indicates greater independence	Pre and post
	Functioning status using Lawton-Brody Instrumental Activities of Daily Living Scale	4-point scale. Higher scores indicate greater independence and better functional ability	Pre and post
Carers			
Carer burden	Carer burden using Zarit Burden Interview	0 to 88 points; higher scores indicate severe burden	Pre and post
Data associated with costs of intervention	Data collected via a proxy resource utilisation questionnaire informed by existing questionnaires such as Resource Utilisation in Dementia (RUD-Lite)	This will be assessed via participants’ case report form and discussion with the researcher including utilisation of primary and secondary healthcare services usage and informal carer time	Post-only

### Nested process evaluation

As part of the study process evaluation, qualitative data will be generated from semi-structured interviews or focus groups conducted by the researcher at 4 months.

A sample of participants receiving home care and their carers (*n* = 12 dyads) will be invited to participate in semi-structured interviews. Family carers will be given the option to have a separate interview, and paid care for the person with dementia will be offered.

Home care professionals (*n* = 12), and home care managers (*n* = 4) will be invited to take part in an interview or a focus group (in pairs of 2–3 staff per provider organisation). We will adopt blended approaches, incorporating a combination of face-to-face interviews (using a Dictaphone) and online interviews/focus groups (via Zoom/Teams software), which will be video and/or audio-recorded and transcribed verbatim.

We will use a pre-designed topic guide, developed in conjunction with our PPI group consisting of key open-ended questions. Participants will be asked to feed back about various aspects of their experiences to inform a potential future trail including receiving or delivering the nutrition intervention, identifying priority areas, the delivery process, how the approach worked, the barriers, facilitators, and aspects that could be improved. For all participants, we will collect demographic data from home care records, including age range, gender, ethnicity, living arrangements, and dementia diagnosis.

Documentary analysis will be undertaken from a review of care plans of people with dementia (at baseline; during preceding 4-month period and at 4 months) using a checklist of key data, e.g. food and drink intake, weight recording, costs.

A bespoke document will be used to collect data on intervention fidelity and dose. This document will be completed by home care professionals, informal carers, or healthcare professionals, or via standardised recording in care plans. Implementation fidelity will be guided by a revised version of Carroll et al.’s conceptual framework [49].

### Feasibility economic component

To estimate the associated costs, data will be collected on the use of health and social care services and support provided by family carers and friends, using a questionnaire adapted from and informed by existing tools such as the Resource Utilisation in Dementia-Lite Version (RUD-Lite) [50]. Data will be gathered from home care records, family carer dyads, and home care professionals.

Specifically, family carers will be asked about the number of hours they spend providing informal care. Data collected on resource use will relate to accessing healthcare, including hospital admissions, ambulance call-outs, length of hospital stay, personal social services, prescription data, and oral nutrition supplements.

This resource use will be costed as per conventional practice via the use of administrative data sets such as NHS Reference Costs, Personal Social Services Research Unit (PSSRU) Costs of Health and Social Care, and the NHSBSA Drug Tariff. Where relevant, missing cost and health-related quality of life data will be estimated using multiple imputation. Levels of missing data per cost category will also be reported.

### Study progression criteria

The findings from this feasibility study will be assessed against key progression criteria from the mixed methods process evaluation data to inform whether to proceed to a future larger trial. In terms of recruitment, as part of the screening process, we will identify the expected number of family carer dyads potentially eligible from each participating organisation’s database and the conversion rate of those who consented to participate [51], i.e. if we can recruit more than 10 dyads from 100 eligible dyads.

Regarding attrition rate, we will aim for a dyad drop-out rate of less than 20%, along with an assessment of the reasons for such loss.

Success for intervention adherence will be determined if there is more than 80% staff adherence to the intervention schedule (assessed via action logs). The intervention and trial procedures will be considered acceptable if there is data completeness of over 80% for each outcome measure and data collection method. The feasibility study could help with defining what adherence and engagement mean in the context of the future nutrition intervention trial. Determining progression criteria is seen as an essential element in assessing the success of a feasibility study [51].

Table 5 outlines the study progression criteria. We will use a traffic light system as a method to define targets for progression: green = progress, amber = amend, red = stop, in combination with data from the process evaluation. This system and findings will be monitored and reviewed by the Project Management Group and reported to the Project Steering Group for input interpretation and input [52].

**Table 5:** TOMATO progression criteria

Domain	Proceed with RCT (Green)	Consider possible improvements (Amber)	Do not proceed (Red) (Red)
Recruitment	≥ 10% of eligible participants enrolled	5–9% of eligible participants enrolled	< 5% of eligible participants enrolled
Retention	≥ 80% of participants retained at follow up	50–79% of participants retained at follow-up	< 50% of participants retained at follow-up
Adherence	≥ 80% staff adherence to the intervention schedule (adherence is defined as setting at least four actions (as documented in the monthly action logs) to do things differently in response to using the resources).	50–79% of staff who adhere to the intervention schedule	< 50% of staff who adhere to the intervention schedule

### Data analysis

#### Quantitative

We will conduct quantitative data analysis primarily using descriptive statistics, including frequencies, means, and percentages with 95% confidence intervals (CIs) (using SPSS v26.0). Categorical data will be presented as frequencies and percentages.

Specifically, we will describe participants’ demographic characteristics and outcome scores collected at baseline and at 4 months using suitable summary statistics. We will also report the proportion of missing data for each outcome measure. We will calculate the differences between baseline and 4-month follow-up, with 95% CIs, for all outcome scores listed in Table 4. We will assess and estimate key progression indicators (using the traffic light system as in Table 5) and obtain stakeholder input to support interpretation of findings and decisions regarding proceeding to a larger trial [52]. These indicators will include recruitment rates, the proportion of homecare staff adhering to the intervention schedule (defined as setting at least four actions over the 4-month intervention period, documented in the monthly action logs), and the proportion of participating clients for whom the outcome measure questionnaire booklet was completed.

We will analyse resource use data collected during the final 1-month follow-up period. Cost will be estimated based on the most recent PSSRU Unit Costs of Health and Social Care [53], and the NHS Drug Tariff for medication costs [54]. Where the specific type of medication is uncertain, we will apply an average cost across all plausible options.

A CONSORT flow diagram will present the flow of participants through the study [37]. The number of participants approached, eligible, consented and enrolled and retained at follow-up will be reported along with reasons for withdrawal or dropout where possible. We will summarise and report rates and patterns of missing questionnaire data to inform the selection of outcomes and administration strategy for a full trial. A statistical analysis plan will be developed and reviewed by the Chief Investigator and Project Management Group prior to commencing analysis.

#### Qualitative

Interview data will be transcribed verbatim, anonymised, and analysed using framework analysis (familiarisation, identifying a thematic framework, indexing charting, mapping and interpretation), including components of feasibility and NPT [39, 55]. Findings will be discussed with the PPI Group to guide interpretation. Researcher memos will be employed throughout the research, including interviews and focus groups, and these memos will be referenced during analysis.

NVivo Pro 20, a qualitative data analysis (QDA) computer software package will be used to facilitate the data analysis.

### Data management

Data will be managed using the Edge platform, the Local Portfolio Management System (LPMS), to capture TOMATO feasibility trial data and support the delivery and maintenance of the study, or an electronic database management system like REDCap, both of which comply with GCP requirements for data management.

All participants enrolled will be assigned unique participant identifiers. Data will be collected in paper or electronic format with various file formats including Microsoft Word, Portable Document Format (PDF), or Microsoft XLSX, based on preference. Electronic files will be transferred using a secure Bournemouth University (BU) file transfer system. All electronic files and the pseudonymisation details will be password-protected and stored on restricted-access folders on a BU secure server accessible only to authorised members of the study team.

All data collected on paper case report forms will be transferred securely using a Study Lockable Document Wallet or in a Travel Secure Rucksack with a lock (using a code or padlock). A delegated research team member will enter and upload participant anonymised data onto the local LPMS system, accessed only by authorised personnel. All paper copies will be stored in a secure BU site filing locker in a timely manner. All data will be archived in line with Bournemouth University’s information security policies and the archiving standard operating procedures.

As the study involves the collection and sharing of data from participating home care providers, risk management processes, including a Data Protection Impact Assessment (DPIA) and a Data Management Plan (DMP), will be in place to address and reduce any associated risks of the intervention and data security.

The TOMATO project team (Project Management Group), led by the chief investigator (JM), will hold monthly meetings to assess and monitor progress, review risks, and mitigate any problems arising. The TOMATO Project Steering Group (PSG) will provide independent oversight for the study.

All person identifiable data will be managed, stored and protected in accordance with the UK General Data Protection Regulation (UK GDPR) and the Data Protection Act 2018 (DPA 2018). Bournemouth University as the sponsor of the study will maintain an oversight of the study as per Good Clinical Practice (GCP) guidelines and the UK Policy Framework for Health and Social Care Research (2017). This includes monitoring of the study records to ensure adherence to GCP.

### Safety reporting

Considering the adverse effects of malnutrition for people living with dementia, the intervention has low risk, as it will help to spot the signs of poor nutrition and provide support for those in receipt of care by staff from the participating home care provider. No serious adverse events (SAEs) are anticipated from participating in this study, although older people living with dementia would be expected to have a higher rate of intercurrent illnesses, such as infections impacting on attrition or falls leading to hospitalisation. We will report adverse events (AEs) and loss to follow-up, monitor for capacity to consent with deteriorating health, and continue to follow up where possible.

To determine the safety of the intervention, all AEs and SAEs and their possible relation to the intervention will be carefully monitored and documented, with classification as either unrelated or related to the intervention. Home care professionals will be trained to document AEs and report back to the research team within a set period of time. All AEs will be followed up until it has been determined that the study intervention is not the cause, and the event has been resolved or a decision has been made for no further follow-up.

The research team will notify the sponsor (BU) within 24 h of any SAEs that are related to the intervention. The Health Research Authority Research ethics committee will be notified within 15 days of the chief investigator becoming aware of the SAE using the non-CTIMP safety report to REC form.

## Discussion

This study will determine the feasibility of our nutrition intervention to improve nutritional care for people with dementia receiving home care, which will inform the development and implementation of a future pilot RCT, should this be feasible. The feasibility study will measure resource use, identify key drivers of associated costs, and provide preliminary information for a future trial. This will be balanced against the costs of hospitalisation and the need for NHS resources associated with adverse events. Through the research, feasibility and acceptability data will be provided to understand the barriers and enablers for implementation. This knowledge will help guide implementation to facilitate greater uptake, adoption and spread into policy and practice in a future study.

Home care professionals are uniquely positioned to play a significant role in supporting people living with dementia. While previous RCTs [24, 25] have shown positive effects on quality of life and delayed nutritional decline through nurse- or multidisciplinary team-delivered nutrition interventions for older adults at home, evidence on interventions delivered specifically by home care professionals remains limited. The TOMATO study will address this gap and contribute valuable insights into the context-specific challenges faced by home care professionals in delivering nutritional care to people living with dementia who are in receipt of home care.

This study has potential far-reaching implications, as identifying nutrition-related health risks and intervening at an early stage for people with dementia living at home can help reduce deterioration in mental and physical health and wellbeing. As such this means there is increased potential for improved quality of life, maintenance of independence, reduced burden for family and home carers and increased likelihood of remaining at home for as long as possible. The study will produce new outputs, including new data, training and toolkit materials and as a feasibility study will be tested to improve the nutritional care of people with dementia living at home and receiving home care.

Additionally, by providing training to home care staff, our work will build on and extend existing skills and support the acquisition of new knowledge, approaches and attitudes, access to resources and to support from health care provision. We anticipate that this will help support home care staff to adopt a strong commitment to the provision of nutritional care for people living with dementia, recognising the important role they can play in this.

## Supplementary Information

Additional file 1

Additional file 2

Additional file 3.

## Data Availability

Plans for data sharing will be completed before the end of the study, and where applicable, a process for requesting access will be made accessible and discoverable.

## Abbreviations

AE: Adverse Event
APEASE: Acceptability, Practicability, Effectiveness/cost-effectiveness, Affordability, Safety/side-effects, Equity
BCTs: Behaviour Change Techniques
BCW: Behaviour Change Wheel
BMI: Body Mass Index
COM-B: Capability, Opportunity and Motivation Behaviour
CONSORT: CONsolidated Standards of Reporting Trials
CPD: Continuing Professional Development
DEMQOL: Dementia Quality of Life
EQ-5D-5L: 5-level EuroQoL-5 Dimension version
FAST: Functional Assessment Staging Test
GCP: Good Clinical Practice
HRA: Health Research Authority
HRQL: Health-Related Quality of Life
HCRW: Health and Care Research Wales
NIHR: National Institute for Health and Care Research
NPT: Normalisation Process Theory
PPI: Patient and Public Involvement
PSSRU: Personal Social Services Research Unit
REC: Research Ethics Committee
REDCap: Research Electronic Data Capture
RE-AIM: Reach, Effectiveness, Adoption, Implementation, and Maintenance framework
RCT: Randomised Controlled Trial
SAE: Serious Adverse Event
SPIRIT: Standard Protocol Items: Recommendations for Interventional Trials
SPSS: Statistical Package for the Social Sciences
TOMATO: Nutrition and Dementia at Home
UKHCA: United Kingdom Homecare Association
