# Supplementary figures and images for: An intervention to provide nutritional care for people living with dementia at home receiving home care (TOMATO): study protocol for a single-arm feasibility study
Source: Pilot Feasibility Stud. 2025 Nov 21;11:146. doi: 10.1186/s40814-025-01722-5 (PMC12639643; doi:10.1186/s40814-025-01722-5)

**Additional File 3: TOMATO Logic Model**


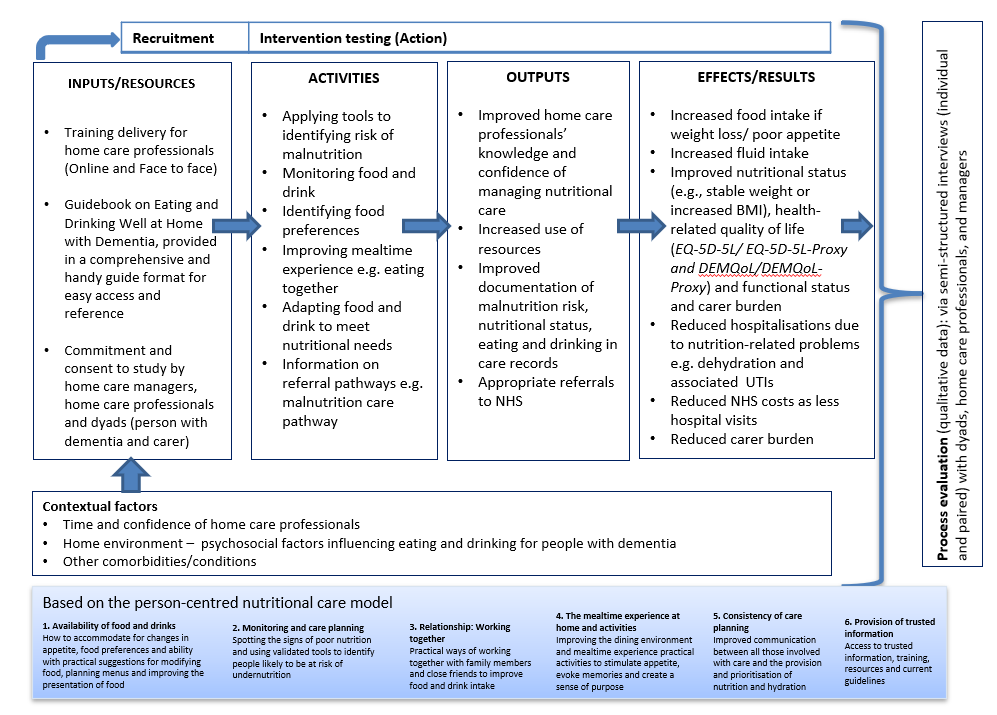

Supplement: Supplementary file 3 — Additional file 3. [file 40814_2025_1722_MOESM3_ESM.docx]
